# Achieving pregnancy safely for HIV-serodiscordant couples: a social ecological approach

**DOI:** 10.7448/IAS.20.2.21331

**Published:** 2017-03-08

**Authors:** Haneefa T Saleem, Manjulaa Narasimhan, Julie A Denison, Caitlin E Kennedy

**Affiliations:** ^a^ Center for Global Health Engagement, Uniformed Services University of the Health Sciences, Rockville, MD, USA; ^b^ Department of Reproductive Health Research, (includes the UNDP/UNFPA/UNICEF/WHO/World Bank Special Programme – HRP), World Health Organization, Geneva, Switzerland; ^c^ Department of International Health, Social and Behavioral Interventions Program, Johns Hopkins Bloomberg School of Public Health, Baltimore, MD, USA

**Keywords:** Safer conception, serodiscordant couples, social ecological models, fertility, HIV, reproductive health

## Abstract

The recognition and fulfilment of the sexual and reproductive health and rights (SRHR) of all individuals and couples affected by HIV, including HIV-serodiscordant couples, requires intervention strategies aimed at achieving safe and healthy pregnancies and preventing undesired pregnancies. Reducing risk of horizontal and vertical transmission and addressing HIV-related infertility are key components of such interventions. In this commentary, we present challenges and opportunities for achieving safe pregnancies for serodiscordant couples through a social ecological lens. At the individual level, knowledge (e.g. of HIV status, assisted reproductive technologies) and skills (e.g. adhering to antiretroviral therapy or pre-exposure prophylaxis) are important. At the couple level, communication between partners around HIV status disclosure, fertility desires and safer pregnancy is required. Within the structural domain, social norms, stigma and discrimination from families, community and social networks influence individual and couple experiences. The availability and quality of safer conception and fertility support services within the healthcare system remains essential, including training for healthcare providers and strengthening integration of SRHR and HIV services. Policies, legislation and funding can improve access to SRHR services. A social ecological framework allows us to examine interactions between levels and how interventions at multiple levels can better support HIV-serodiscordant couples to achieve safe pregnancies. Strategies to achieve safer pregnancies should consider interrelated challenges at different levels of a social ecological framework. Interventions across multiple levels, implemented concurrently, have the potential to maximize impact and ensure the full SRHR of HIV-serodiscordant couples.

## Introduction

For HIV-serodiscordant couples who desire a child, access to acceptable and appropriate healthcare services and programmes, including for the prevention of vertical and horizontal HIV transmission and assisted reproductive technologies to improve HIV-related infertility, is important for achieving pregnancy safely. While anxiety around transmission of HIV to partners and children does exist, there is evidence that the desire for pregnancy can outweigh these concerns [1]. The recognition and fulfilment of the sexual and reproductive health and rights (SRHR) of all individuals, including HIV-serodiscordant couples, therefore includes the need for interventions to support safe and healthy pregnancies, for those who desire children. While this paper is focused on individuals and couples desiring a child, it is recognized that for those who do not desire a child or wish to space their pregnancies, long-acting contraception and other appropriate healthcare services and counselling are required. Creating, building and strengthening an enabling environment grounded in a human rights framework to support choice, autonomy and agency of individuals and couples can enable them to achieve their SRHR.

A set of interrelated, multi-level factors affect the capacity of HIV-serodiscordant partners to access relevant and appropriate information, counselling and services related to both HIV and SRHR interventions. Improved access to such information and services can in turn be beneficial for improving SRHR outcomes as well as other aspects of health and wellbeing. A social ecological framework provides a comprehensive model for understanding the multiple and interacting determinants of SRHR for HIV-serodiscordant couples and suggests interventions at multiple levels that can influence individual and couple behaviours and outcomes [2].

While different variants of social ecological frameworks have been proposed [2–4], they generally embody four guiding principles. First, they recognise the multiple influences on health behaviours and outcomes. Second, they posit that these influences interact across these different levels. Third, they require a focus on specific health behaviours and outcomes, identifying which factors are most likely to influence the specific behaviour or outcome at each level of the framework. Lastly, social ecological frameworks suggest that interventions that address factors at multiple levels are likely to be more effective than those that address only one level.

In this commentary, we explore challenges and opportunities for achieving pregnancy safely for HIV-serodiscordant couples globally using a social ecological framework developed by Crankshaw et al. for understanding HIV risk behaviour in the context of supporting their fertility goals [2]. We draw from and expand on components of this framework, which include the following levels: (1) the structural domain that encompasses the sociopolitical, economic and cultural context; gender norms and ideologies; health systems and the legal and policy environment, (2) the individual determinants, such as HIV status, clinical health and fertility desires and (3) couple-level determinants that are mediated by the relationship context, which includes gender power and communication. These levels interact and shape the behaviours of couples and their desired fertility outcomes [2]. The discussion is organized based on these different levels of influence within the following topical areas: achieving pregnancy safely; facilitated disclosure of HIV status; fertility counselling and support, for HIV-serodiscordant couples desiring a child, including those who may be experiencing infertility; and policy and financial considerations.

## Discussion

### Achieving pregnancy safely

Achieving a pregnancy safely includes both antiretroviral therapy (ART)-based and behavioural safer conception strategies that can be adopted by HIV-serodiscordant couples who desire a pregnancy. These strategies also support the elimination of mother-to-child transmission of HIV by preventing HIV transmission to an uninfected woman in a serodiscordant relationship and, ultimately, to the children of serodiscordant couples [5].

#### ART-based strategies

Suppression of the HIV viral load of the partner living with HIV to undetectable levels is effective in reducing HIV transmission [6–8]. In the absence of HIV suppression, for example before ART initiation or during the first six months of ART when residual HIV transmission risk has been shown to persist [9], oral pre-exposure prophylaxis (PrEP) can effectively serve as a bridge for reducing HIV transmission until ART-related viral suppression is achieved [10].

#### Behavioural strategies

Behavioural strategies available for safer conception include manual vaginal insemination [11,12] and timed intercourse without a condom around the most fertile period of the woman’s menstrual cycle [13].

Serodiscordant partners often need counselling and support to achieve skills to perform behaviours that support achieving a safer pregnancy, which at the individual level require adequate knowledge and skills of ART-based and behavioural safer conception strategies. However, many women and men living with HIV have insufficient knowledge and skills to correctly and consistently use ART and/or oral PrEP [14–16]. Research has also shown that strategies to support safe pregnancy, such as manual vaginal insemination, are often little known and/or unaffordable to people living with HIV [12,17–20]. In the structural domain, healthcare providers often lack clinical guidance, training and support in counselling serodiscordant couples on how to achieve pregnancy safely, or how to integrate training into individualized counselling messages [18,21–23]. Additionally, provider training in SRHR service delivery is not always effective due to underlying health system constraints [24]. Lack of knowledge and training among healthcare providers may help to explain reports of stigma and discrimination experienced within the healthcare system by people living with HIV who desire a child, including coercive and forced sterilization [25,26] that are human rights violations [18,27,28]. Stigma and discrimination can also affect the pregnancy desires, choices and outcomes for HIV-serodiscordant couples. For example, stigma and discrimination by communities and healthcare providers may discourage serodiscordant couples from having children despite fertility desires, or it may prevent them from accessing safer conception services or safe pregnancy care once they do succeed in getting pregnant [27,29].

Social norms and expectations around gender within the structural domain shape interactions between serodiscordant couples in the relationship context and individual self-efficacy to engage in crucial conversations around fertility desires and achieving pregnancy safely. In some settings, unequal gender power dynamics within couples lead men to play a dominant role in decisions about pregnancy [18]. Engaging men in discussions with their partners on issues of safer conception and safer pregnancy may help change these dynamics and promote risk reduction strategies that support safe pregnancies.

Community-based interventions aimed at sensitising partners, families and neighbours on conception and childbearing for women and men living with HIV could decrease stigma, promote equality and inclusion, and build broad societal norms and structures in support of the realization of the reproductive goals and rights of serodiscordant couples [30,31]. Furthermore, interventions within the health system to enable the achievement of safe pregnancies include providing adequate training to healthcare providers and integrating SRHR and HIV services. Integration in particular has been shown to be both beneficial and feasible, with positive effects on HIV and SRHR outcomes [21,32].

### Facilitated disclosure of HIV status

Women and men living with HIV can benefit from mutual and voluntary disclosure of their HIV status to their partners, and this has been associated with improved ART initiation, adherence and retention [33–35], which are important components that support serodiscordant couples to achieve pregnancy safely. Disclosure of HIV status may also be needed for serodiscordant couples to use PrEP for viral suppression or prevention of HIV acquisition, timed intercourse or manual vaginal insemination. However, many people living with HIV, particularly women, may be hesitant to disclose their HIV status due to fear of stigma, discrimination and particularly violence [36,37]. To reduce the risk of violence resulting from HIV status disclosure to a partner, HIV and SRHR policymakers, programme managers and healthcare providers could work towards developing and implementing novel approaches for safer disclosure, including assessing for partner violence and engaging peer-counsellors and communities of people living with HIV [38–40]. There is also a need to explore programmatic and clinical approaches that could be used in cases of non-disclosure of HIV status when disclosure could increase harm [41].

### Fertility counselling and support

Women and men living with HIV may have increased risk of infertility, including the inability to become pregnant or impregnate a partner or to maintain a pregnancy, due to various behavioural and physiological mechanisms [42]. Serodiscordant couples may choose to consistently use condoms during sexual intercourse to prevent HIV transmission to the uninfected partner and prevent pregnancy [42]. Physiological conditions, such as advanced HIV disease progression, interactions with ART, higher frequency and severity of genital tract infections and acquisition of other sexually transmitted infections, have all been shown to impair fertility in women and men living with HIV [43,44]. Additionally, women living with HIV may have increased susceptibility to gynaecological diseases and disorders, such as cervical dysplasia and pelvic inflammatory disease, which may reduce fertility or complicate the course of a pregnancy [43].

Social norms regarding parenthood can contribute to stigma and discrimination of serodiscordant couples who are unable to conceive or maintain a pregnancy [27,45]. Infertility in women has been associated with increased risk of experiencing intimate partner violence in low- and middle-income countries [46]. For serodiscordant couples who experience infertility or seek advice on fertility options, strategies exist that range from simple fertility awareness methods to more advanced approaches associated with assisted reproductive technology, such as *in vitro* fertilization [47,48] and sperm washing followed by intrauterine insemination or intracytoplasmic sperm injection [49]. These innovative interventions have revolutionized concepts of family and human reproductive potential. However, the availability and costs of assisted reproduction may prevent some couples from accessing these services. Adoption is also a means to fulfil the desire for a child. Serodiscordant couples desiring a child through adoption rather than pregnancy should be supported in this choice. Through an integrated HIV and SRHR model of care, healthcare providers could support serodiscordant couples by conducting fertility assessments, counselling individuals and couples on their fertility treatment options and linking couples to more specialized assisted reproduction or adoption services [42].

### Promoting legislation, policies and funding streams to support HIV-serodiscordant couples achieve pregnancy safely

Promoting laws and policies that are supportive of the fertility desires of people living with or affected by HIV can contribute to increased health, wellbeing and realization of their reproductive goals and rights. Improved access to ART, PrEP, contraceptive methods and choice and acceptable, affordable assisted reproductive technologies continues to be a challenge globally for many individuals and couples, including for HIV-serodiscordant couples. The International Conference of Population and Development Programme of Action (ICPD PoA) defines reproductive health as “a state of complete physical, mental and social well-being, and not merely the absence of disease or infirmity, in all matters relating to the reproductive system and to its functions and processes” (Paragraph 7.2) [50]. The ICPD PoA also discusses sexual health, “the purpose of which is the enhancement of life and personal relations, and not merely counselling and care related to reproduction and sexually transmitted diseases”. It further defines reproductive rights as embracing ''certain human rights that are already recognized in national laws, international human rights documents and other consensus documents. These rights rest on the recognition of the basic right of all couples and individuals to decide freely and responsibly the number, spacing and timing of their children and to have the information and means to do so, and the right to attain the highest standard of sexual and reproductive health. It also includes their right to make decisions concerning reproduction free of discrimination, coercion and violence, as expressed in human rights documents'' (Paragraph 7.3) [50]. Importantly, the UN Committee on Economic, Social and Cultural Rights (CESCR) in General Comment 22 recognises that “people living with HIV/AIDS are more likely to experience multiple discrimination” and that “States must reform laws that impede the exercise of the right to sexual and reproductive health, including in relation to HIV status and transmission” and recommends adoption of “appropriate legislative, administrative, budgetary, judicial, promotional and other measures to ensure the full realization of the right to sexual and reproductive health” (Paragraph 45) [50,51].

Unfortunately, limited funding for HIV and SRHR services, staff shortages, unreliable supplies of essential medication and screening equipment and missed opportunities for healthcare provider training are barriers to providing comprehensive and holistic SRHR services, including those for achieving and supporting a safe and healthy pregnancy for serodiscordant couples [18]. Cost-saving approaches, such as task shifting of HIV- and SRHR-related tasks from doctors to trained nurses or shifting fertility counselling from doctors and nurses to community health workers or peer-counsellors, could also be adopted without reducing the quality of care received by people living with HIV [52,53].

### Adopting a multi-level approach to achieving safe pregnancy among HIV-serodiscordant couples

**Figure 1.**
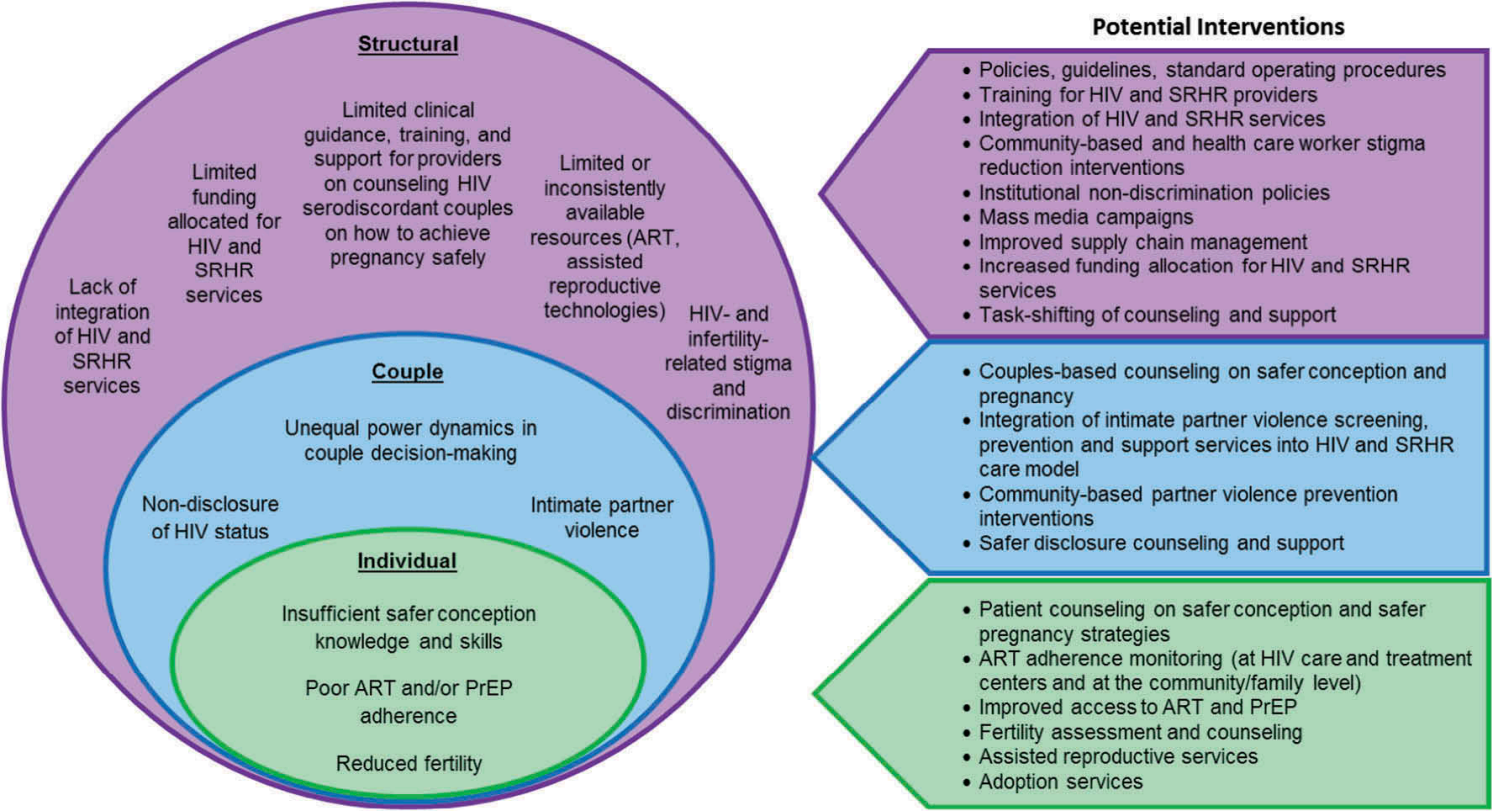
Social ecological framework of challenges to achieving pregnancy safely among HIV-serodiscordant couples and potential interventions to address challenges. ART: antiretroviral therapy; HIV: Human immunodeficiency virus; PrEP: pre-exposure prophylaxis; SRHR: sexual and reproductive health and rights

Within each level, there are clearly opportunities for distinct intervention to mitigate the challenges serodiscordant couples face with achieving pregnancy safely. The value of social ecological frameworks, however, is the opportunity to examine interactions between levels and how interventions at multiple levels influence individual behaviours and outcomes. Social norms and expectations around gender shape interactions between couples and individual self-efficacy to engage in crucial conversations around disclosure, fertility intentions and safe pregnancy. Global financial inequalities shape access to assisted reproductive technologies, ART and PrEP and influence individual-level financial resources that can facilitate access or adherence to these interventions. Interventions focused at each level have traditionally been emphasized. However, in order to effectively innovate in supporting serodiscordant couples to achieve their reproductive goals and rights, national and local health governing and implementing bodies may need to intervene across the individual, couple and structural domains with comprehensive, multi-level intervention packages. Figure 1 presents potential interventions that could be implemented to foster safer conception and safer pregnancy among serodiscordant couples. The successful implementation of multi-level interventions will require multisectoral collaboration between government, civil society and the private sector, and the meaningful involvement of communities of women and men living with HIV to ensure that their priorities and needs inform policies and guidelines and shape HIV and SRHR services [54].

## Conclusions

It is the choice of each individual and couple to determine if they desire a pregnancy, and if so, the size of their family unit and the timing of when to have, or not have, a child. Studies from a range of geographic and economic contexts affirm that individuals and couples affected by HIV, regardless of setting, continue to desire children. Some women living with HIV or women in serodiscordant relationships may feel pressured to have a child due to a partner, family or gender norms within the community or may be deterred from getting pregnant because of stigma, family and community concerns for the health of a potential child. However, there have been significant changes in the lives of people living with HIV in the past decade. This includes the rapid expansion of ART and the development of PrEP, as well as WHO recommendations to offer immediate ART to all individuals living with HIV and oral PrEP to HIV-uninfected individuals at substantial risk. Individuals and couples affected by HIV who desire a child can now exercise their right to attempt pregnancy, found a family and feel an integral part of their communities. Strategies to achieve safer pregnancies should consider interrelated barriers and opportunities across multiple levels of a social ecological framework, implemented concurrently, in order to maximize impact and ensure the full SRHR of HIV-serodiscordant couples.
